# Population parameters of the orange mud crab *Scylla olivacea* (Herbst, 1796) from the Sundarban mangrove forest in Bangladesh

**DOI:** 10.1016/j.heliyon.2021.e06223

**Published:** 2021-02-12

**Authors:** Muhammad Abdur Rouf, Sheik Istiak Md Shahriar, Al-Hasan Antu, Md Noman Siddiqui

**Affiliations:** a Fisheries and Marine Resource Technology Discipline, Khulna University, Khulna, 9208, Bangladesh; b Department of Aquaculture, Hajee Mohammad Danesh Science and Technology University, Dinajpur, 5200, Bangladesh

**Keywords:** Growth, Recruitment, Mortality, Exploitation, *Scylla olivacea*, Bangladesh

## Abstract

Population parameters of orange mud crab (*Scylla olivacea*) were estimated, aiming to determine sex ratios, carapace width-body weight (CW-BW) relationships, asymptotic width (CW_∝_), growth coefficient (K), mortality (Z, M, and F), recruitment and level of exploitation (E) in the Sundarban mangrove forest, located in the south-western part of Bangladesh. Year-round samples were collected using hook-lines and baited traps, the population parameters were measured from CW frequency data using FiSAT-II analyzer. The study showed that the overall male and female sex ratio was 1:0.66, revealing a male domination in the study area. The CW-BW relationship indicated that the increment rate in the BW of the male crabs (b = 3.06, R^2^ = 0.98) were higher than that of female (b = 2.62; R^2^ = 0.98) *S. olivacea*. The b value differed significantly (P < 0.006) from isometric growth (b = 3) where males exhibited positive and females exhibited negative growth allometry. Estimated CW_∝_ for male and female were 164 mm and 152 mm along with K values 0.90 yr^−1^ and 0.76 yr^−1^, respectively. Total mortality (Z) was 2.67 yr^−1^ and 1.57 yr^−1^, natural mortality (M) was 0.98 yr^−1^ and 0.90 yr^−1^ and fishing mortality (F) was 1.69 yr^−1^ and 0.67 yr^−1^ for male and female, accordingly. Recruitment of both sexes exhibited a bimodal recruitment pattern where young population occurs continuously throughout the year and a major peak of recruitment for males was observed from November to January and for female it was from February to April. The estimated exploitation rate (E) for male (0.63) was higher than the female (0.43) where the E for male exceeded the maximum permissible limit (E = 0.50). A remarkable range of fishing pressure at lower size classes was revealed in this study and thus framing minimum legal size is crucial for effective management of the population.

## 1 Introduction

Mud crab (genus *Scylla*) has four globally recognized species i.e. *Scylla serrata*, *Scylla olivacea, Scylla paramamosain* and *Scylla tranquebarica* (Keenan et al., 1998). Among these species, *S. serrata* was considered the most common in Bangladesh. After having a long controversy on the taxonomic clarification of genus *Scylla*, it has now been confirmed, based on its morphometric, meristic and genetic analysis, that *S. olivacea* is the common mud crab species harvested from Sundarban mangrove swamps (Rouf et al., 2016; Sarower et al., 2017). However, the species of genus *Scylla* are extensively distributed in the tropical waters of the Indo-pacific regions along with the coast of the Indian Ocean (Keenan et al., 1998). The coastal ecosystem of Bangladesh including its canals, rivers, streams, tributaries and estuaries provides muddy substrate, which plays a vital role for the habitat, shelter and nursery ground of commercially important mud crab species including *S. olivacea* (Acharya and Kamal, 1994). The usual niche of genus *Scylla* is intertidal and sub-tidal regions of mangrove wetlands. More explicitly, juvenile crabs are mainly found in the intertidal mangrove zone whereas adults are mostly found in the sheltered inshore and/or deeper sub-tidal estuarine areas (Ward et al., 2008). Since spawning occurs in the deep sea, abundance of larval population escalates with the increase of distance from the coast to sea (Sara, 2010).

In Bangladesh, *S. olivacea* has gained importance as a potential aquatic resource export after shrimp (*Penaeous monodon*) and simultaneously, has ranked as second highest foreign currency earning crustacean species (DoF, 2016). Due to high demand in the world market, the intense harvest of mud crab occurs year-round from the wild. Moreover, crab cultures in coastal ponds fully depend on wild seed stock, collected from the Sundarban adjacent areas, due to the absence of fully functional mud crab hatcheries. Capturing this huge quantity of juvenile and adult mud crabs are ultimately resulting in intense pressure on the wild crab population. Therefore, estimation of population parameters and exploitation levels of this economically viable *S. olivacea* stock from the south-western coast of Bangladesh warranted high importance for proper utilization and management strategies.

Carapace width (CW) frequency distribution, carapace width-body weight (CW-BW) relationships and growth parameters have great significance in estimating the population size of a unit stock having same gene pool. In addition, these parameters help to adopt effective management strategies for the sustainable and judicious exploitation of aquatic resources (Bal and Rao, 1984). Carapace width frequency distribution of a species supports to construct different size classes that are attributed to various approximate age groups which finally help to have clear concept on spawning of that particular species (King, 1995). Moreover, CW-frequency data have been widely used in determining age and evaluating growth parameters of crustaceans because it is a quick, more accurate and reliable method (Viswanathan et al., 2016).

Most of the studies regarding mud crab in Bangladesh are focused on taxonomic confirmation (Rouf et al., 2016; Sarower et al., 2017), fattening technology (Kamal et al., 2007), culture and bio-economics (Ferdoushi and Xiang-Guo, 2010), recruitment and growth performance (Zafar et al., 2006) and stock assessment (Chantarasri, 1994). So far, only one study was done in Bangladesh by Zafar et al. (2006) on the population dynamics of *S. serrata*, which particularly focused on the south-eastern part of Bangladesh. Therefore, there is a gap of information on the population parameters of mud crabs in the south-western part. Considering the above mentioned importance, this study aimed to estimate the key population parameters including sex ratio, carapace width-weight (CW-BW) relationship, asymptotic carapace width (CW_∝_), growth co-efficient (K), recruitment, mortality (Z, M and F), exploitation level (E) and virtual population analysis (VPA) of *S. olivacea.* These data are necessary for formulating proper management and conservation policies for mud crab resources in Bangladesh.

## 2 Materials and methods

**Figure 1:**
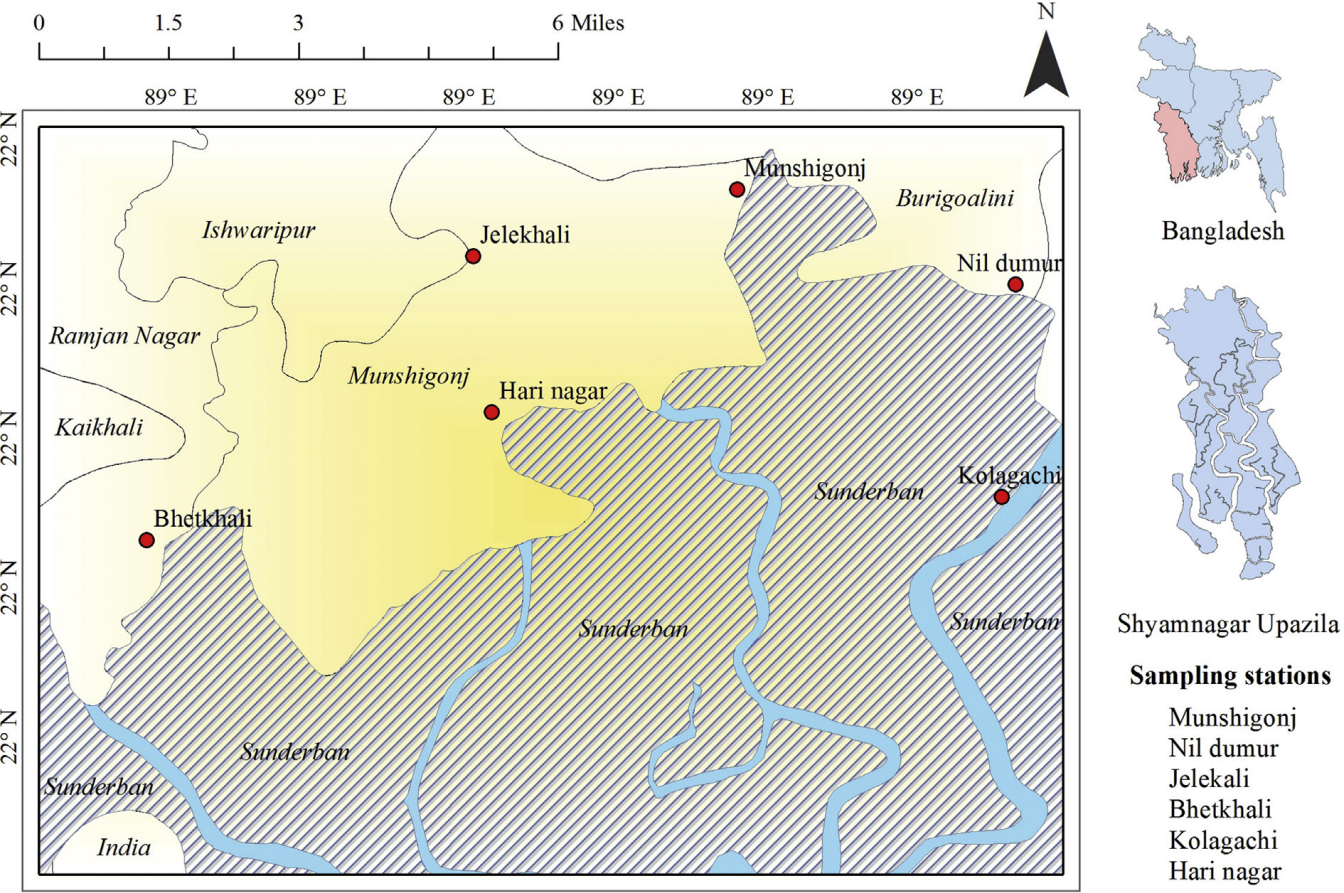
Location of the sampling stations of *S. olivacea* adjacent to Sundarban mangrove, Bangladesh.

*Study area and sampling:* The study was carried out considering six (6) sampling stations at Shyamnagar Upazila (21°36′-22°24′N and 89°00′-89°19′E) adjacent to Sundarban mangrove forest, Bangladesh. The locations of the sampling stations are shown in Figure 1. Sampling was done fortnightly over a 12 months' period from July 2017 to June 2018 using two types of gear. Hooks and lines are mostly used in this area for crab harvesting. A total of 15 hooks baited with small fish (*Cuchia*) were set at each station during flood and ebb tide. Baited basket traps were also placed with same bait at a distance of 25–30 m in shallow water approximately for 6 h.

*Data collection:* A total of 3191 specimens of *S. olivacea*, comprising 1969 males and 1222 females, were collected during the study period. The species and its sex were identified based on the morphological characteristics following Keenan et al. (1998). The CW was measured using digital slide calipers to nearest 0.05 mm and weighed using an electronic balance close to 0.1 g.

*Data analysis:* Sex ratio was determined dividing the total number of females by the total number of males of the respective size class. The CW-BW relationship of *S. olivacea* was described for each gender using the method potency equation W = aL^b^ (W = BW in g, L = CW in mm, a and b are constants). The observed CW and BW values of individual crabs were log-transformed and subjected to linear regression analysis (log BW = log a + b∗log CW). The significance of coefficient b was tested using the Student's t-test at 5% significance level. One-way analysis of variance (ANOVA) was used to analyze the relationship between CW and BW.

Asymptotic carapace width (CW_∝_) and growth co-efficient (K) of the von Bertalanffy equation for growth in terms of CW were estimated by means of Electronic Length Frequency Analysis (ELEFAN-I) incorporating in the FiSAT-II (FAO-ICLARM Stock Assessment Tools) using the following formula;

(1) $${\text{CW}}_{\text{t}}={\text{CW}}_{\text{α}}\left(1-{\text{e}}^{-\text{K}\left(\text{t}-{\text{t}}_{\text{o}}\right)}\right)$$

where, t = age of the species (yr), CW_t_ = mean carapace width at age t (mm), t_0_ = the hypothetical age at which the CW is zero, K = growth co-efficient (yr^−1^). According to Pauly and Munro (1984), the growth performance index (Φ) of *S. olivacea* derived from K and CW_∝,_ is as follows;

(2) $$\Phi =\text{Log K}+2\;\text{Log }{\text{CW}}_{\text{α}}$$

Total mortality (Z) was determined considering length converted catch curve method stated by Beverton and Holt (1956) considering a standard habitat temperature of 25 °C. Natural mortality (M) was projected using the empirical relationship given by Pauly (1980); fishing mortality (F) was estimated by subtracting natural mortality (M) from total mortality (Z). Mortality (Z, M and F) was measured in yr^−1^. Exploitation level (E) was the portion of total mortality due to fishing and it was estimated according to Gulland (1971);

(3) E=F/Z

Recruitment pattern was obtained by backward projection on the CW axis of a set of CW-frequency data using FiSAT-II software. The probabilities of capture were assessed through the ‘probability of capture’ routine and the estimated CW structured virtual population analysis was also calculated using the FiSAT-II analyzer. The experiment was approved by the Animal Ethics Committee of Khulna University, Bangladesh. The approval number is KRAEC-2020/08/11.

## 3 Results

The CW-frequency data of each sampling day was classified into 5 mm size intervals and the size frequency distribution of males and females are shown in Table 1. The minimum and maximum carapace width (CW) was 16.1 mm and 164 mm, weighted 2.35g and 610.5 g for male; whereas for female CW ranged from 17.3 mm to 161 mm, weighted from 3.78 g to 587.12 g. Mean and standard deviation (SD) of carapace width (CW) and body weight (BW) depending on sex and life stages of *S. olivacea* are shown in Table 2. The results of one way ANOVA indicated that BW with class range 70–120 mm, 121–170 mm and 16–170 mm showed significant difference (p < 0.05) between male and female mud crabs. The highest number of male and female crabs was found in 111–115 mm and 101–105 mm size class, respectively.

**Table 1:** Size class distribution of *S. olivacea* in Sundarban mangrove of Bangladesh.

Juveniles (<70 mm)			Sub-adults (71–120 mm)			Adults (>120 mm)		
CW (mm)	Male (No.)	Female (No.)	CW (mm)	Male (No.)	Female (No.)	CW (mm)	Male (No.)	Female (No.)
16–20	5	17	71–75	45	22	121–125	153	83
21–25	10	22	76–80	47	45	126–130	108	67
26–30	19	23	81–85	70	34	131–135	72	57
31–35	30	28	86–90	74	45	136–140	50	32
36–40	34	14	91–95	107	44	141–145	21	31
41–45	41	18	96–100	135	62	146–150	16	15
46–50	27	17	101–105	164	117	151–155	15	5
51–55	29	21	105–110	194	116	156–160	12	3
56–60	27	12	111–115	217	115	161–165	5	1
61–65	28	23	116–120	182	107	**-**	-	-
66–70	30	26	**-**	-	-	**-**	-	-

**Table 2:** Mean and standard deviation (SD) of carapace width (CW) and body weight (BW) depending on sex and life stages of *S. olivacea*.

Life stages	Male (mean ± SD)		Female (mean ± SD)	
	CW (mm)	BW (g)	CW (mm)	BW (g)
Juveniles	54.18 ± 9.53	36.25 ± 15.43	54.17 ± 9.51	36.16 ± 13.15
Sub-adults	101.71 ± 12.97	254.70 ± 99.10	101.42 ± 13.48	179.33 ± 61.43
Adults	130.07 ± 7.35	487.21 ± 51.76	130.94 ± 7.55	389.0 ± 63.62
Overall	102.05 ± 24.5	279.05 ± 156.6	99.57 ± 26.8	199.91 ± 126.3

**Figure 2:**
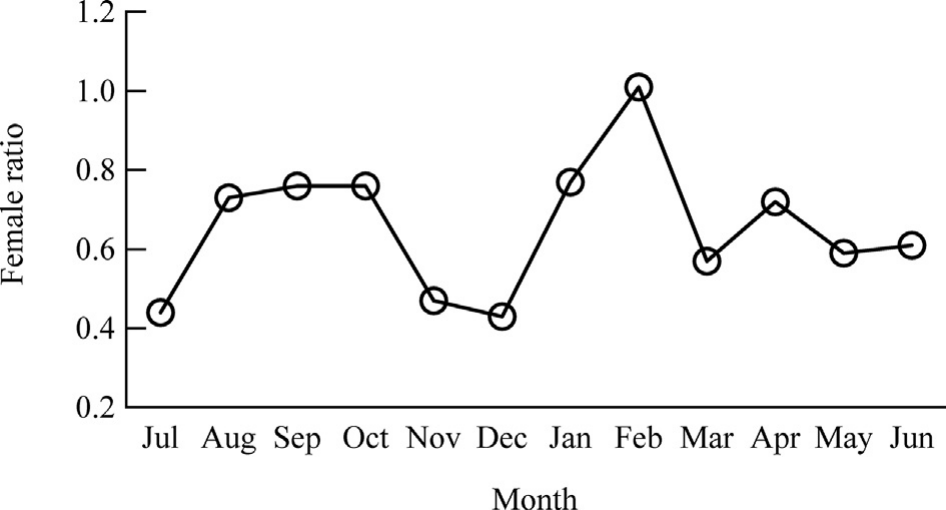
Month-wise sex ratio of female considering standard male ratio 1.0.

A total of 3191 crabs were captured from the study area where male and female proportion was 61.70% and 38.30%. The overall sex ratio (male:female) was 1:0.66. The month-wise male-female ratio addressed that the number of females largely decreased in July, November and December and mostly appeared again in August–October and February (Figure 2). However, male dominated sex ratio was observed in all the months except February.

**Figure 3:**
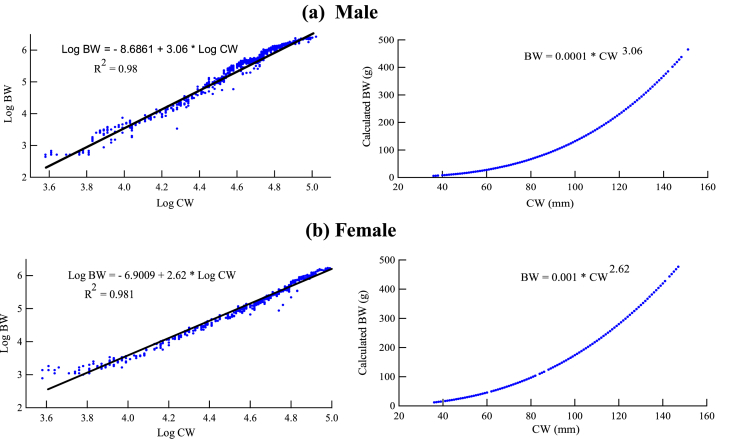
The relationship between the carapace width (CW) and body weight (BW) of (a) male and (b) female *S. olivacea* in the Sundarban mangrove waters of Bangladesh.

A strong positive correlation (r > 0.99) was observed between CW and BW of *S. olivacea* (Table 3). The CW-BW relationship for male and female was estimated as: BW = 0.0001CW^3.06^ (R^2^ = 0.98; P = < 0.0001; n = 1969) and BW = 0.001CW^2.62^ (R^2^ = 0.98; P = < 0.0001; n = 1222), respectively (Figure 3). The b value differed significantly (P < 0.006) from isomerism (b = 3) where males exhibited positive and females exhibited negative growth allometry. The estimated b also indicated higher increment rate of BW in male than female of *S. olivacea* (Figure 3).

**Table 3:** CW-BW relationship of *S. olivacea* sampled from Sundarban mangrove of Bangladesh.

Sex	Size (N)	a	b	r	R^2^	Allometry	P value
Male	1969	0.0001	3.06	0.99	0.980	+ve	<0.001
Female	1222	0.001	2.62	0.99	0.981	-ve	<0.001

**Figure 4:**
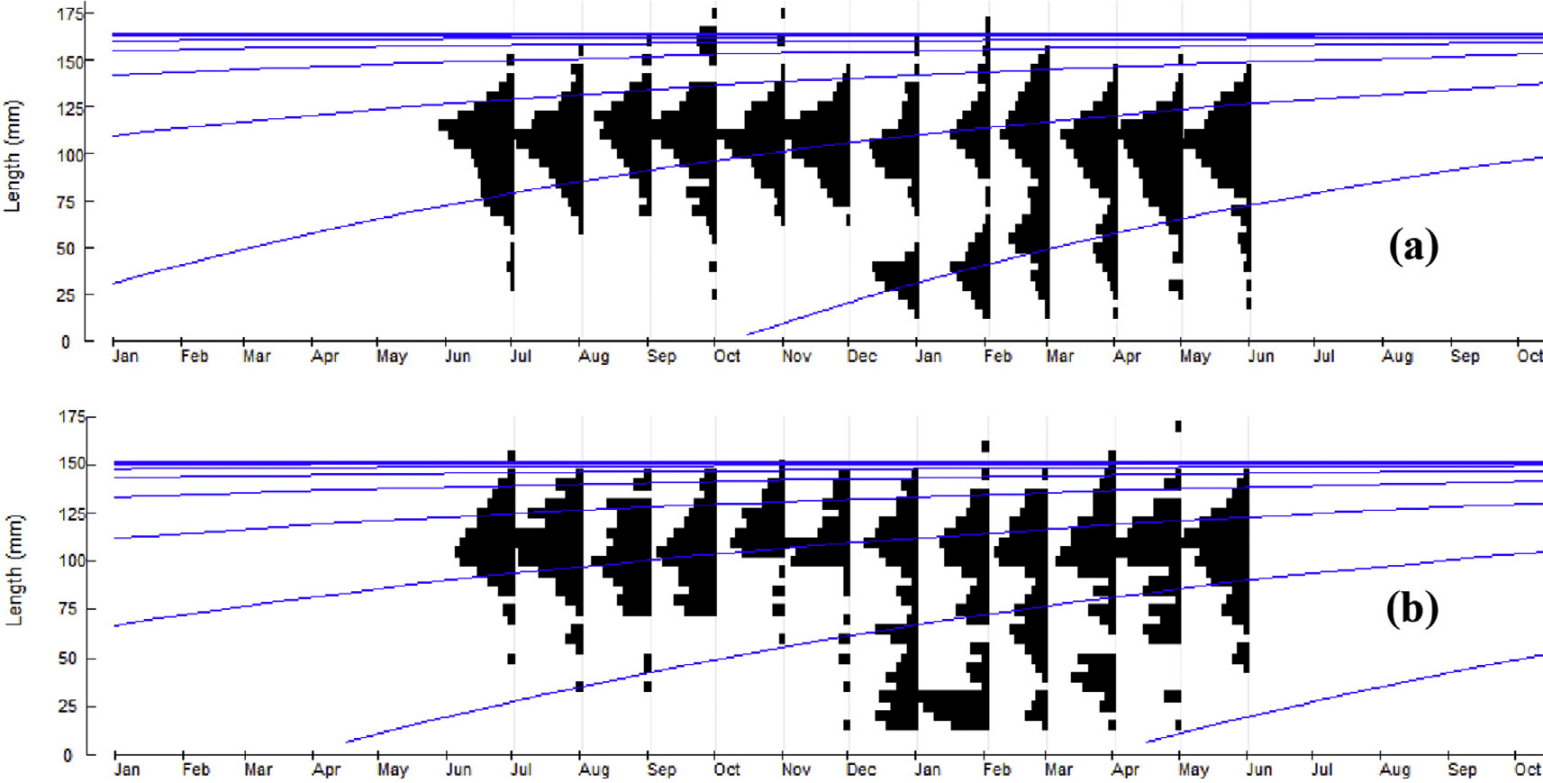
Von Bertalenffy growth curves of (a) male and (b) female *S. olivacea* drawn over their restructured length distribution.

The von Bertalenffy growth function (VBGF) fitted to size increment data led to estimate asymptotic carapace width (CW_∝_) 164 mm and 152 mm along with growth co-efficient (K) as 0.90 yr^−1^ and 0.76 yr^−1^ for male and female (Table 4 and Figure 4). The estimated growth performance index (Φ) for male was 4.34 and female was 4.21.

**Table 4:** Population parameters of *S. olivacea* collected from the Sundarban mangrove, Bangladesh.

Sex	Parameters							
	CW_∝_ (mm)	K (yr^−1^)	M (yr^−1^)	F (yr^−1^)	Z (yr^−1^)	E	E_max_	Status
Male	164	0.90	0.98	1.69	2.67	0.63	0.50	E > E_max_
Female	152	0.76	0.90	0.67	1.57	0.43	0.50	E < E_max_

**Figure 5:**
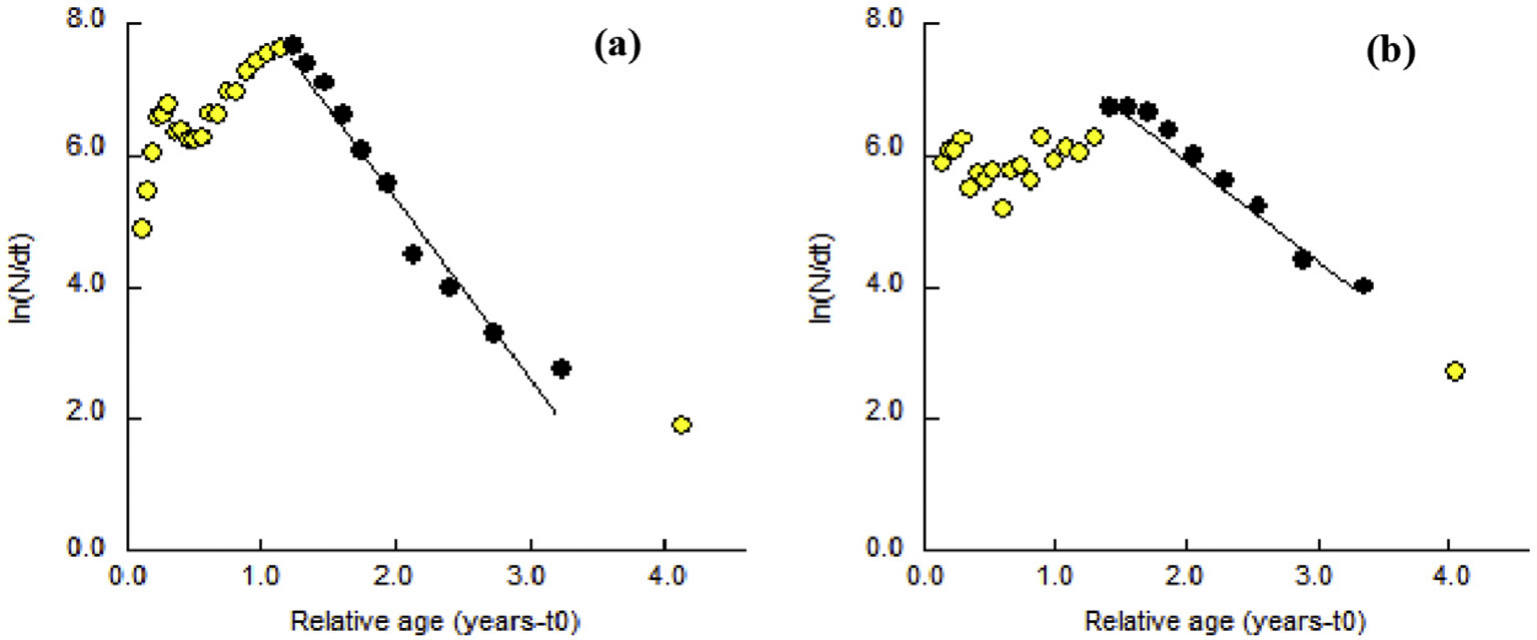
Length converted catch curves for (a) male and (b) female *S. olivacea* of Sundarban Mangrove of Bangladesh (The filled dots represent the points used in calculating through least square liner regression and the open dots represent the point either not fully recruited).

The estimated Z value was 2.67 yr^−1^ and 1.57 yr^−1^ (Figure 5), M was 0.98 yr^−1^ and 0.90 yr^−1^ and F was 1.69 yr^−1^ and 0.67 yr^−1^ for male and female, respectively (Table 2). The M was higher than F in female, but the opposite scenario was observed in male. The estimated E value for male and female *S. olivacea* was 0.63 and 0.43, respectively. It indicated that the E of male exceeded the maximum permissible limit of exploitation (E = 0.50) where female E was lower than the permissible limit (Table 4).

**Figure 6:**
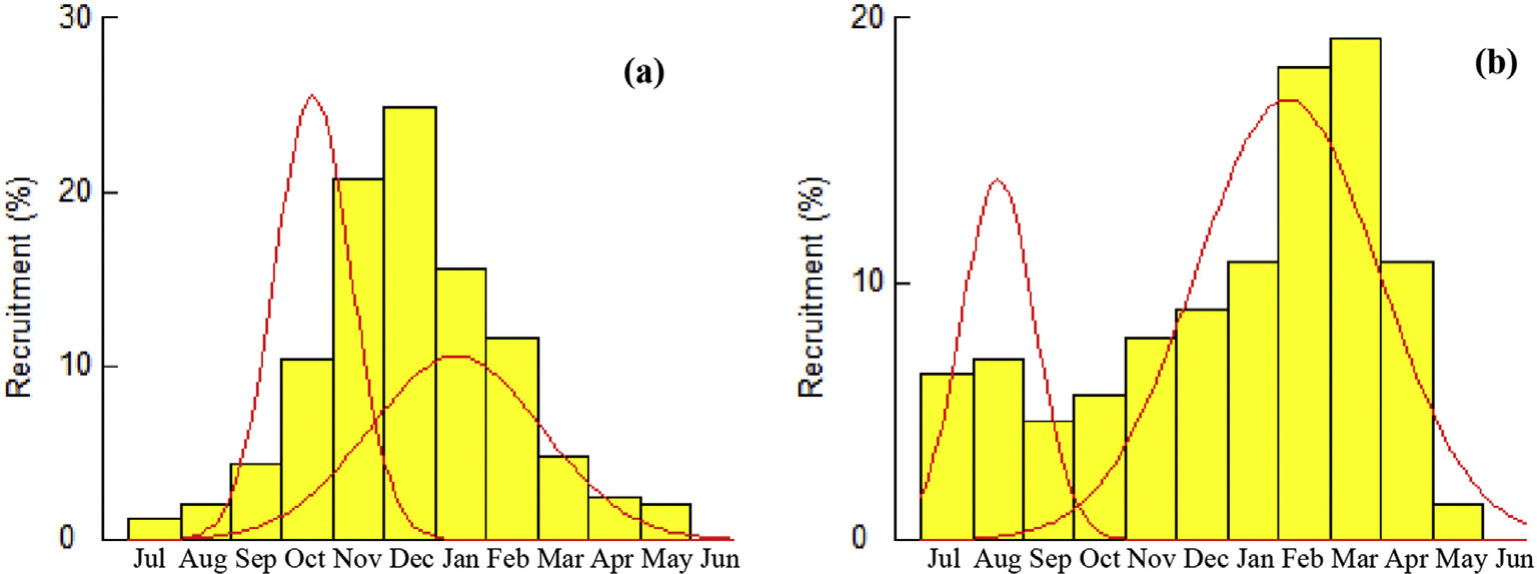
Annual recruitment patterns of (a) male and (b) female *S. olivacea* sampled from Sundarban mangrove of Bangladesh.

The recruitment of *S*. *olivacea* in the study area occurred throughout the year (Figure 6). The major peak for male was observed from November to January (Figure 6a). In case of female, the major recruitment peak was observed from February to April and the minor one occurred in between September and October (Figure 6b).

**Figure 7:**
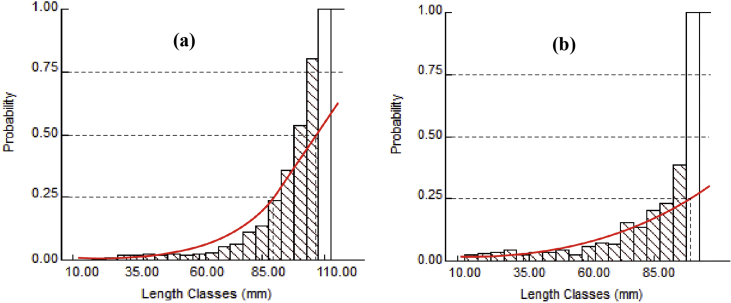
Probability of capture based on length (CW) classes of (a) male and (b) female *S. olivacea*.

**Figure 8:**
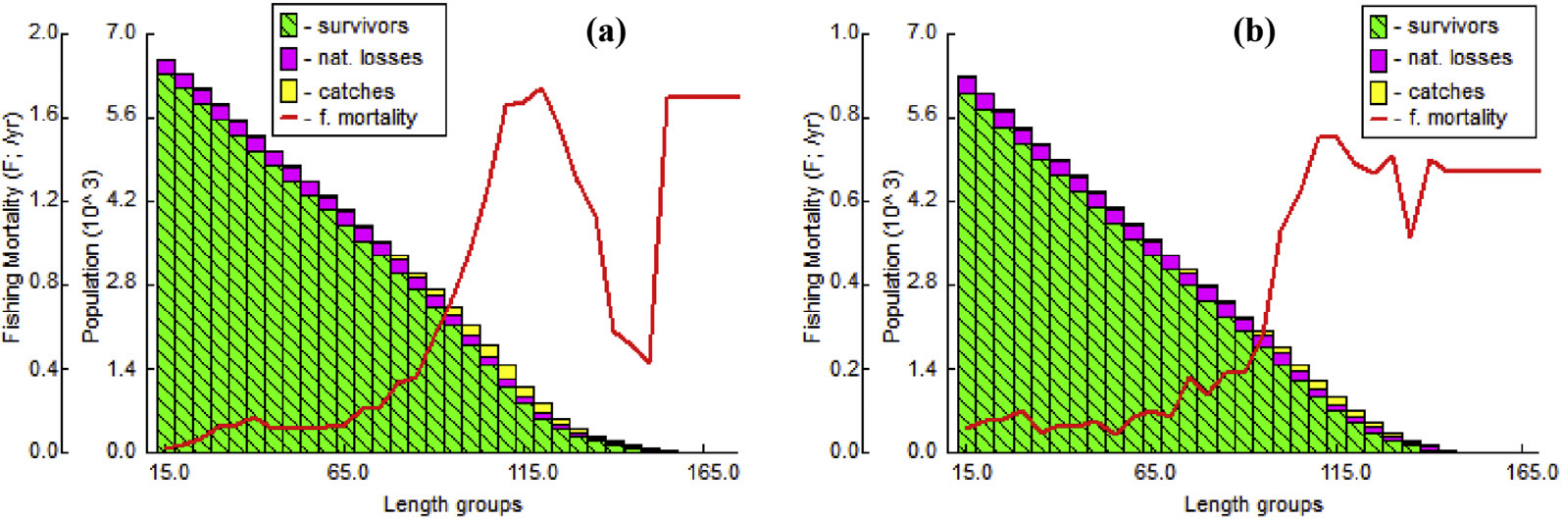
Length (CW) based virtual population analysis for (a) male and (b) female *S. olivacea* collected from the Sundarban mangrove, Bangladesh.

The analysis of capture probabilities for male revealed that 25% of 88.79 mm CW, 50% of 106.66 mm CW and 75% of 124.53 mm CW were vulnerable to the gears, whereas, in female it was 99.25 mm CW (25%), 130.97 mm CW (50%) and 162.70 mm CW (75%) (Figure 7). The virtual population analysis (VPA) employed to estimate the extent of mortality on various size classes of *S. olivacea* (Figure 8). The fishing pressure on *S. olivacea* was maximum in the size of 85–120 mm for males and 90–135 mm for females.

## 4 Discussion

The present study was conducted to determine several important population parameters based on CW-frequency data of *S. olivacea* sampled from Sundarban mangrove forest adjacent areas of Bangladesh.

Size frequency distribution of a species helps to know which specific catch group (i.e. juvenile, sub-adult or adults) is more vulnerable to fishing gears. This study revealed that the *S. olivacea* stock recruitment occurs over 20 mm of CW. The smallest CW of this mud crab recruitment occurs at 35mm, 45mm, and 47mm CW in Philippine, India, and Malaysia respectively (Viswanathan et al., 2019; Waiho et al., 2016; Walton et al., 2006). The largest CW of male crab in this study was 164 mm (mean 102.05 ± 24.5 mm) whereas the CW was 148 mm (mean 104.33 ± 77.4 mm) in India (Viswanathan et al., 2019) and 134 mm CW (mean 90.4 ± 12.66 mm) in Malaysia (Waiho et al., 2016). The CW of *S. olivacea* caught from Sundarban was quite high; nevertheless, the mean value is comparatively low. Greater difference between the highest CW and mean CW indicates the overexploitation of this species (King, 1995).

The sex ratio of a specific crustacean species is imperative as it helps to comprehend the population structure and reproductive status of that species (Overton and Macintosh, 2002; Wardiatno and Mashar, 2010). The overall recorded male and female ratio in present study was 1:0.66 and evinced the existence of male domination over female in the population. The male and female sex ratio was 1:0.87 in the Pichavaram mangroves of India (Viswanathan et al., 2019) and 1:0.77 in Malaysian mangrove swamp (Jirapunpipat, 2008). Relatively lower percentage of female especially in sub-adult population class size (Figure 8) is found in the study areas. The lower percentage of females in a crab population is related with their ecological niche i.e. intertidal burrows and/or the migration towards deep sea for breeding purposes (Sara, 2010). In addition, food availability, fishing pressure and genetic variability also affect the sex ratio of a specific species (Araújo and Lira, 2012).

In regression analysis, the coefficient of determination (R^2^) for male and female were very close to 1 (Table 3), suggesting a high degree of positive relation prevails between the CW and BW of the collected samples. The exponential (b) value is a robust indicator of fish growth especially when the value of b is equal to 3; the fish is supposed to be growing in its entire dimension i.e. length, width and weight. If b < 3, the adult fish species in a unit stock possess more elongated body form than the juveniles (Froese, 2006). On the contrary, when b > 3, the adults are increasing more in width than the length and tends to become thicker in body shape and heavier in weight (Bolger and Connolly, 1989). There are several factors that cause variation in slope (b) likely water quality parameters, food availability, access to food, sample size and length ranges (Mommsen, 1998; Ighwela et al., 2011). However, the estimated exponential (b) value for female (2.62) was lower than the isometric growth value (b = 3); showing negative allometry. Conversely, positive allometric growth (b > 3) was recorded for male samples. Similar growth pattern of *S. olivacea* reported from Malaysia and India (Table 5). However, the length-weight relationship might be inconsistent across the Portunidae family due to exploitation and/or differences in physical factors likely salinity, temperature, pH, rainfall and dissolved oxygen (Waiho et al., 2016). The estimated relationship also infers that males are slightly heavier than that of females having same CW might be due to the allometric enlargement of male chelae with sexual maturation (Miyasaka et al., 2007) and this tendency of males to acquire relatively higher weight among Portunids is observed in many studies (Abdel-Razek et al., 2006). The relationship parameters for genus *Scylla* from different published literatures are shown in Table 5.

**Table 5:** Parameters of CW-BW relationship of Portunid crabs from different parts of the world.

Location	Species	Sex	a	b	R^2^	Source
Bangladesh (South-west coast)	*S. olivacea*	Male	0.0001	3.06	0.980	Present study
		Female	0.001	2.62	0.981	
Malaysia (Taiping)	*S. olivacea*	Male	0.00001	3.548	0.971	Waiho et al. (2016)
		Female	0.001	2.558	0.982	
Malaysia (Setiu)	*S. olivacea*	Male	0.00006	3.278	0.982	
		Female	0.002	2.489	0.975	
India (Pichavaram)	*S. olivacea*	Male	3.035	3.804	0.962	Viswanathan et al. (2016)
		Female	2.925	3.592	0.933	
Bangladesh (South-east coast)	*S. serrata*	Male	0.251	2.870	0.991	Zafar et al. (2006)

The actual growth of crustacean in nature is hard to measure as it depends on molting. The frequency of molting increases at early stage of a crab and continue till its sexual maturity and the frequency of molting decreases as the age increases (Kodama et al., 2005). Besides, growth parameters contrast between same species, and likely depend on niche, environmental factors, biological variability and disease susceptibility of the species (Sara, 2010).

The present findings on the CW_∝_ (male = 164 mm and female = 152 mm) and K (male = 0.90 yr^−1^ and female = 0.76 yr^−1^) of *S. olivacea* from Bangladeshi water attain greater significance as there is no such investigations carried out on this particular species. The CW_∝_ of same species in other Asian countries including India and Malaysia are similar. The CW_∝_ of male and female *S. olivacea* were 148.05 mm and 138.8 mm, respectively in Pichavaram mangroves India (Viswanathan et al., 2018). Similarly, the CW_∝_ of common *S. olivacea* was 154.82 mm in Setiu wetland areas, Malaysia (Zaidi et al., 2011). The growth is considered higher when the K is > 1. The overall growth rate of *S. olivacea* in this study was slower as the K value for both sexes were less than 1 (Sparre and Venema, 1998). In addition, the K value of male *S. olivacea* was found greater than female in most of the studies (Table 6). Matured female crabs use most of their energy for reproduction rather than somatic growth and resulting lower growth, but males can grow faster due to its higher energy utilization from food to bolster metabolic processes (Kembaren and Ernawati, 2012). Growth performance index (Φ) is a valuable parameter to select species for culture; amidst the closely related species which has higher Φ is selected for aquaculture to gain higher biomass and more profit in short time as well. The index value for male mud crabs was greater than that of the females (Table 6). Higher K and Φ value in male causes comparatively faster growth and higher CW_∝_.

**Table 6:** Comparison of VBGF parameter of different of species of Portunid crabs.

Location	Species	Sex	CW_∝_ (mm)	K (yr^−1^)	Φ (phi)	Source
Bangladesh	*S. olivacea*	Male	164.00	0.90	4.34	Present study
		Female	152.00	0.76	4.21	
India	*S. olivacea*	Male	148.15	0.76	4.22	Viswanathan et al. (2018)
		Female	138.80	0.64	4.09	
Malaysia	*S. olivacea*	Combined	154.82	0.53	4.10	Zaidi et al. (2011)
Indonesia	*S. serrata*	Male	211.5	1.38	4.79	Sara (2010)
		Female	210.2	0.83	4.56	
Bangladesh	*S. serrata*	Male	105.50	0.28	3.50	Zafar et al. (2006)
		Female	152.00	0.36	3.60	
Indonesia	*Portunus pelagicus*	Male	152.04	0.93	4.33	Hamid et al. (2015)

Recruitment of both sexes exhibited a bimodal recruitment pattern where young crabs are added to the population continuously throughout the year in the study area with a major recruitment peak for males from November to January and for females from February to April (Figure 6). Similarly, Viswanathan et al. (2019) reported major recruitment of *S. olivacea* from December to March in Pichavaram mangrove, India. They also reported that larval development and succession of juveniles take place within 3–4 months after breeding. Considering this time frame, projected breeding peak of *S. olivacea* in the present study would be around September and October. Monthly sex ratio records in current study showed a significant deviation of females in October and December. Migration for spawning after breeding to the offshore may be responsible for this deviation (Figure 2). However, in *Scylla* spp., the recruitment is reported throughout the year from Bangladesh (Zafar et al., 2006) as well as from other countries (Moser et al., 2005; Walton et al., 2006). Nevertheless, crustacean recruitment might be influenced by the male and female ratio, spawning frequency, predation, fishing pressure, disease, various physio-chemical factors importantly temperature, salinity, nutrients, current and velocity (Caputi et al., 2010; Green et al., 2014).

The fishing mortality (F) was higher in male than female, here; it occurs as males grow relatively larger in size and are more susceptible to fishing gear. The male of *S. olivacea* in this study were under fishing pressure (F > M) and overexploited (E = 0.63), suggesting over exploitation should be controlled in the study area. Similarly, the stock of *S. olivacea* and *Portunus pelagicus* (Portunid species) are under over-exploitation in India and Thailand (Table 7). Though the size classes for maximum fishing pressure obtained in this study was similar to Viswanathan et al. (2018) but a remarkable range of fishing pressure at lower size classes was observed in the VPA (Figure 8). If this fishing pressure continues at the same rate, it will negatively affect the stock, sex ratio and ultimately breeding success. To protect this crab population, proper management measures such as application of size limit and setting up of crab hatcheries is very important.

**Table 7:** Comparison of mortality (M, F and Z) and exploitation levels (E) of different mud crab species.

Location	Species	Sex	M (yr^−1^)	F (yr^−1^)	Z (yr^−1^)	E	Source
Bangladesh	*S. olivacea*	Male	0.98	1.69	2.67	0.63	Present study
		Female	0.90	0.67	1.57	0.43	
India	*S. olivacea*	Male	0.21	0.76	0.97	0.78	Viswanathan et al. (2018)
		Female	0.37	0.74	1.11	0.66	
Malaysia	*S. olivacea*	Combined	1.11	0.01	1.12	-	Zaidi et al. (2011)
Indonesia	*S. serrata*	Male	2.48	1.20	3.68	-	Sara (2010)
		Female	1.78	0.75	2.75		
Bangladesh	*S. serrata*	Male	0.49	0.35	0.84	0.41	Zafar et al. (2006)
		Female	0.58	.038	0.96	0.39	
Indonesia	*P. pelagicus*	Male	1.09	1.71	2.80	0.61	Hamid et al. (2015)
		Female	0.86	2.09	2.95	0.71	
India	*P. pelagicus*	Male	2.72	2.45	4.54	0.54	Josileen and Menon (2007)
		Female	2.11	1.57	3.03	0.52	

## 5 Conclusion

To sum up, the CW-BW relationship of *S. olivacea* implied that males are quite heavier than that of females whereas the overall growth performances were comparatively slower. Possible spawning season has been obliquely predicted using length-frequency distribution data; however, finding actual spawning season for the *S. olivacea* in Bangladesh depending on real time data needs to be focused where this study can be evident as reference point. Besides that implementation of effective management strategies like catch size restriction, limiting number, efficiency and types of fishing units could be effective to reduce the enhanced fishing pressure on *S. olivacea* in Sundarban mangrove of Bangladesh.

In Bangladesh, crab fishing is officially prohibited in Sundarban and its adjacent areas from January to February. However, attention of national policy makers is necessary to consider the observations reported in this study in order to protect the population and facilitate the local agencies to implement responsible fishing and sustainable utilization of mud crabs in Bangladesh.

## Declarations

### Author contribution statement

Muhammad Abdur Rouf, Shaik Istiak Md. Shahriar: Conceived and designed the experiments; Performed the experiments; Analyzed and interpreted the data; Contributed reagents, materials, analysis tools or data; Wrote the paper.

Al-Hasan Antu: Performed the experiments; Analyzed and interpreted the data; Wrote the paper.

Md. Noman Siddiqui: Analyzed and interpreted the data; Contributed reagents, materials, analysis tools or data; Wrote the paper.

### Funding statement

This research did not receive any specific grant from funding agencies in the public, commercial, or not-for-profit sectors.

### Data availability statement

Data will be made available on request.

### Declaration of interests statement

The authors declare no conflict of interest.

### Additional information

No additional information is available for this paper.
